# Hydroxyurea‐Associated Wunderlich Syndrome in Triple‐Negative Myelofibrosis: A Case Report and Literature Review

**DOI:** 10.1155/crh/5518867

**Published:** 2026-05-21

**Authors:** Veysel Erol, Zeki Güzel, İbrahim Ethem Akgün, Gökhan Ecer

**Affiliations:** ^1^ Department of Hematology, Kahramanmaras Necip Fazil City Hospital, Kahramanmaras, Turkey; ^2^ Department of Pathology, Kahramanmaras Necip Fazil City Hospital, Kahramanmaras, Turkey; ^3^ Department of Radiology, Kahramanmaras Necip Fazil City Hospital, Kahramanmaras, Turkey; ^4^ Department of Urology, Konya Karapinar State Hospital, Konya, Turkey

**Keywords:** drug-induced vasculitis, hydroxyurea, myelofibrosis, spontaneous renal hemorrhage, Wunderlich syndrome

## Abstract

**Background:**

Wunderlich syndrome (WS) is a spontaneous, nontraumatic intrarenal or perirenal hemorrhage most commonly related to renal neoplasms, vascular disorders, infection, cyst rupture, or anticoagulation. Clinical severity ranges from self‐limited bleeding to hemorrhagic shock.

**Case:**

We report a 58‐year‐old woman with triple‐negative myelofibrosis (MF) who was started on hydroxyurea (HU) 500 mg/day and low‐dose acetylsalicylic acid (ASA). On Day 60, she presented with bilateral perirenal and intrarenal hemorrhage confirmed by contrast‐enhanced abdominal computed tomography. ASA was discontinued and bleeding ceased spontaneously. One month later, she represented with recurrent bilateral hemorrhage without ASA exposure and marked neutrophilic leukocytosis. Concomitant purpuric skin lesions, positive antinuclear antibodies (ANA, 1:160), and low C3 levels raised suspicion for a HU‐associated vasculitic process; HU was discontinued. Coagulation parameters at the time of bleeding (INR: 1.2, aPTT: 26.4 s, fibrinogen: 348 mg/dL, and D‐dimer: 0.6 μg/mL) were not consistent with disseminated intravascular coagulation. Despite supportive management, the patient’s condition deteriorated and she died shortly thereafter. The exact cause of death could not be definitively determined.

**Conclusion:**

To our knowledge, this is the first reported case of WS in a patient with MF receiving HU therapy. Clinicians should consider WS in HU‐treated patients presenting with acute flank pain or hematuria and evaluate for potential vasculitic features.

**Summary**
• New Findings
◦ To our knowledge, this is the first reported case of Wunderlich syndrome (WS) occurring in a patient with myelofibrosis receiving hydroxyurea (HU). While HU is widely used as a cytoreductive agent in chronic myeloproliferative neoplasms, its association with spontaneous perirenal or intrarenal hemorrhage has not been described previously. The temporal relationship to HU exposure, vasculitic skin lesions with positive antinuclear antibodies (ANA) and low C3 levels, and recurrence of hemorrhage despite aspirin discontinuation collectively suggest HU‐associated vascular inflammation as a plausible mechanism. Although causality cannot be definitively established, this case expands the spectrum of potential HU‐related complications and underscores the need to consider WS in HU‐treated patients presenting with flank pain or macroscopic hematuria.

## 1. Introduction

Myelofibrosis (MF) is classified within chronic myeloproliferative neoplasms (CMPNs) and is characterized by cytopenias, leukocytosis, splenomegaly, extramedullary hematopoiesis, and risk of leukemic transformation. The identification of the JAK2 V617F mutation has significantly influenced treatment strategies. Depending on clinical features, therapeutic approaches include hydroxyurea (HU), ruxolitinib, interferon, momelotinib, pacritinib, fedratinib, and allogeneic hematopoietic stem cell transplantation (AHSCT) [1].

Wunderlich syndrome (WS) is a rare condition characterized by spontaneous intrarenal or perirenal hemorrhage in the absence of trauma. While some cases follow a self‐limited course, others may progress to hypovolemic shock and death [2]. Herein, we present a case of triple‐negative MF complicated by bilateral spontaneous renal hemorrhage temporally associated with HU therapy.

## 2. Case

### 2.1. Initial Presentation and Diagnosis

A 58‐year‐old woman was referred for evaluation of isolated anemia. She reported night sweats and weight loss. Her only medication was levothyroxine for hypothyroidism. Laboratory results showed a white blood cell (WBC) count of 12.570/μL, hemoglobin (Hb) level of 9.7 g/dL, and platelet (Plt) count of 213.000/μL. Peripheral smear examination revealed occasional dacrocytes without circulating blasts. Mild hyperferritinemia (310 ng/mL) was observed. Abdominal imaging demonstrated significant splenomegaly with a craniocaudal length of 22 cm.

**FIGURE 1:**
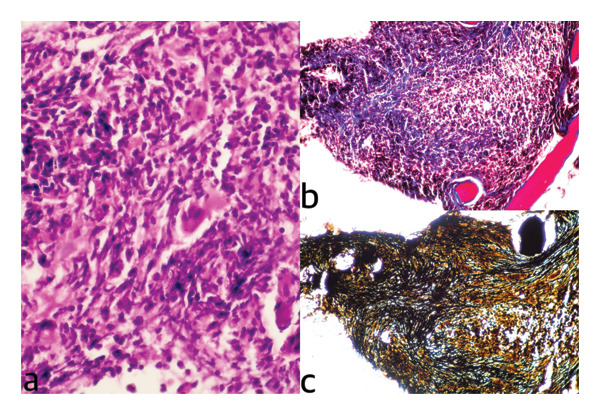
Bone marrow biopsy images. (a) Hypercellular bone marrow with atypical megakaryocytic proliferation (hematoxylin and eosin stain). (b) Masson’s trichrome stain showing focal areas of collagen deposition. (c) Reticulin staining revealing Grade MF‐3 fibrosis.

Bone marrow aspiration yielded a dry tap. Subsequent trephine biopsy demonstrated Grade MF‐3 reticulin fibrosis according to the European Consensus Grading System, confirming the diagnosis of MF (Figure 1). There was no prior history of essential thrombocythemia or polycythemia vera; therefore, the patient was diagnosed with primary (de novo) triple‐negative MF.

Based on age, Hb level below 10 g/dL, leukocytosis, and the presence of constitutional symptoms, she was classified as intermediate‐2 risk according to the Dynamic International Prognostic Scoring System (DIPSS) [1]. Baseline Plt counts were within normal limits (213.000/μL at the time of diagnosis), and there was no documented clinical evidence of Plt dysfunction or bleeding diathesis prior to initiation of HU therapy.

Genetic testing for JAK2 V617F, JAK2 exon 12, CALR, and MPL mutations was negative, and the patient was, therefore, classified as having triple‐negative MF.

### 2.2. Treatment and First Hemorrhagic Episode

**FIGURE 2:**
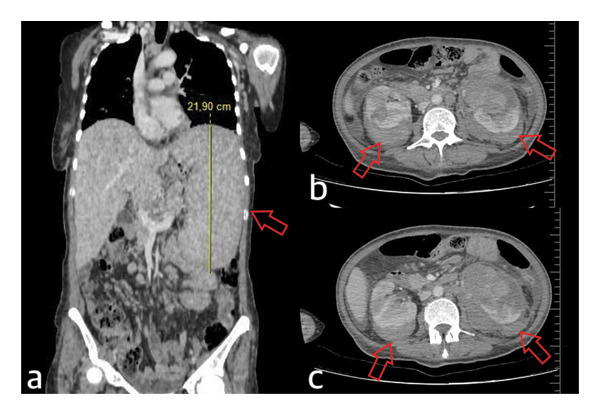
Contrast‐enhanced abdominal computed tomography (CT) images including portal venous phase acquisition. (a) Coronal portal venous phase image demonstrating marked splenomegaly (21.9 cm, arrow). (b‐c) Axial portal venous phase images showing bilateral perirenal and intrarenal hyperdense collections consistent with acute hemorrhage (arrows), without evidence of contrast‐enhancing renal mass, angiomyolipoma, aneurysm, or other detectable vascular malformation.

HU (500 mg/day) and acetylsalicylic acid (ASA, 81 mg/day) were initiated. On Day 60 of therapy, the patient presented with severe bilateral flank pain and massive hematuria. Contrast‐enhanced abdominal computed tomography (CT), including portal venous phase imaging, demonstrated bilateral perirenal and intrarenal hyperdense collections consistent with acute hemorrhage (Figure 2). CT angiography was not performed due to hemodynamic instability at presentation. Ultrasonographic evaluation did not reveal a renal mass, angiomyolipoma, aneurysm, or detectable vascular malformation.

At presentation, the patient was hypotensive (blood pressure: 90/60 mmHg) and tachycardic (heart rate: 108 beats/min). Hb decreased from a baseline value of 9.5 g/dL to 7.1 g/dL. She received intravenous fluid resuscitation and two units of packed red blood cells. No vasopressor support was required. The patient did not require intensive care unit admission and stabilized within 24 h.

Renal function remained preserved during this episode (serum creatinine 0.9 mg/dL compared with a baseline value of 0.8 mg/dL). Urinalysis confirmed gross hematuria without significant proteinuria or active sediment suggestive of glomerulonephritis.

ASA was discontinued. After 24 h of observation, bleeding ceased spontaneously, and the patient achieved hemodynamic stability.

At the time of the hemorrhagic event, coagulation parameters were not consistent with disseminated intravascular coagulation (DIC). The international normalized ratio (INR) was 1.2, and activated partial thromboplastin time (aPTT) was 26.4 s. The fibrinogen level was within normal limits (348 mg/dL), and D‐dimer was 0.6 μg/mL without significant elevation. Plt count was not critically reduced, and peripheral smear did not demonstrate schistocytes or features of microangiopathic hemolysis. These findings did not fulfill laboratory criteria for DIC and argue against consumptive coagulopathy as the underlying mechanism of hemorrhage.

### 2.3. Recurrent Hemorrhage and Vasculitic Findings

One month later, the patient presented with recurrent similar symptoms. Laboratory findings revealed WBC 91.250/μL (neutrophils 83,380/μL), Hb 5.6 g/dL, and Plt 110,000/μL (Table 1). Peripheral smear demonstrated neutrophilic leukocytosis without blasts. Importantly, ASA had not been reintroduced prior to this episode.

**TABLE 1:** Laboratory parameters at diagnosis and during the first and second hemorrhagic episodes.

Laboratory parameter	24 June 2024 (at diagnosis)	25 August 2024 (first hemorrhagic episode: on ASA)	01 October 2024 (second hemorrhagic episode: off ASA)
Creatinine (mg/dL) (0.51–0.95)	0.65	0.7	0.68
LDH (U/L) (0–248)	137	140	299
WBC (× 10^3^/μL) (4.49–12.68)	12.57	11.25	91.25
Neutrophils (× 10^3^/μL) (2–9)	9.49	8.3	83.38
Lymphocytes (× 10^3^/μL) (1.2–3.4)	1.3	1.25	3.11
Monocytes (× 10^3^/μL) (0.25–1)	1.66	1.37	4.59
Hemoglobin (g/dL) (11.5–15)	9.7	7.1	5.6
Platelets (× 10^3^/μL) (150–400)	213	230	110
INR	1.1	1.2	1.09
aPTT (seconds)	25.4	26.4	23.7
D‐dimer (μg/mL)	0.7	0.6	0.8
Fibrinogen (mg/dL) (200–400)	280	348	360

*Note:* LDH, lactate dehydrogenase; WBC, white blood cell count.

Abbreviations: aPTT, activated partial thromboplastin time; INR, international normalized ratio.

During this recurrence, noncontrast abdominal CT confirmed massive bilateral perirenal and intrarenal hemorrhage. Contrast‐enhanced imaging and CT angiography were not performed due to hemodynamic instability. Repeat ultrasonographic evaluation did not reveal an aneurysm or vascular malformation.

**FIGURE 3:**
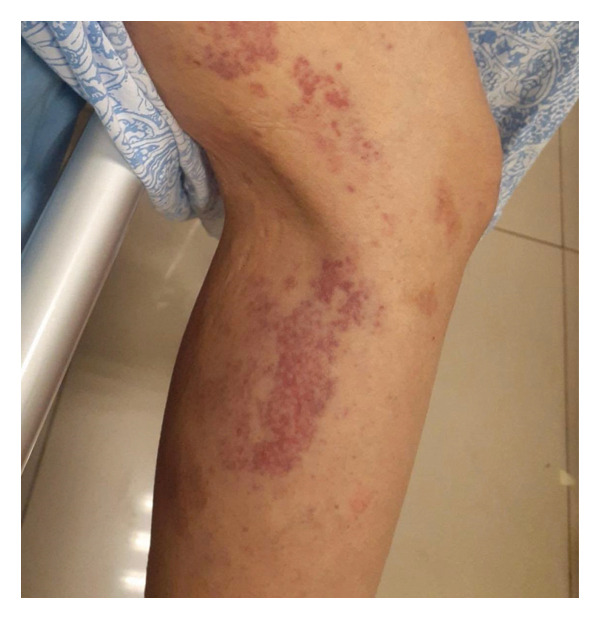
Cutaneous lesions observed after hydroxyurea treatment, consisting of purpuric 1–3 mm macules, some barely palpable, located on the anterior and medial thighs.

During this period, urticarial and petechial skin lesions developed (Figure 3). Antinuclear antibodies (ANA) were positive at a titer of 1:160. Complement C3 levels were decreased (54.6 mg/dL), whereas C4 levels were within normal limits. ANCA testing was negative. A skin biopsy could not be performed due to the patient’s compromised clinical status and increased bleeding risk.

Given the recurrence in the absence of aspirin exposure, exclusion of DIC, absence of structural renal pathology on imaging, and emergence of immunologic abnormalities and cutaneous findings, HU was discontinued due to suspicion of a drug‐associated vasculitic process.

The patient was referred to a tertiary center for possible urological intervention. Conservative management resulted in spontaneous resolution of the hemorrhage.

At the time of recurrence, laboratory findings revealed marked neutrophilic leukocytosis and worsening anemia accompanied by moderate thrombocytopenia (Plt: 110,000/μL). There were no clinical or laboratory signs of active infection. Peripheral smear showed no circulating blasts.

### 2.4. Clinical Deterioration and Death

**FIGURE 4:**
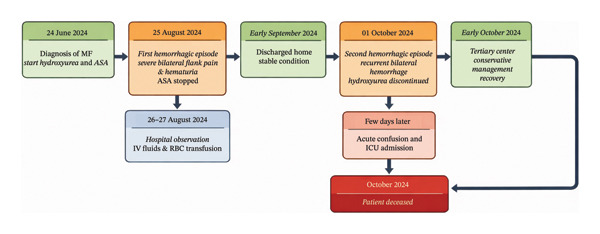
Clinical timeline of the case illustrating the sequence of treatment initiation, hemorrhagic episodes, therapeutic modifications, referral to a tertiary center, and clinical outcome.

Within days of discharge, the patient developed acute confusion followed by a fall and rapid clinical deterioration requiring admission to the intensive care unit. No recurrent renal hemorrhage was documented at that time. Despite advanced life support measures, the patient died. The exact cause of death (e.g., intracranial event, sepsis, multiorgan failure, or another complication) could not be definitively established based on the available medical records. Therefore, a direct causal relationship between the hemorrhagic episodes and death cannot be conclusively determined. The overall clinical course is summarized in Figure 4.

## 3. Discussion

WS is a rare syndrome characterized by spontaneous renal hemorrhage. Lenk’s triad—flank pain, palpable mass, and hypovolemic shock—is observed in approximately 20% of the cases [3, 4]. The most common etiologies include angiomyolipoma, renal cell carcinoma, vascular diseases, and anticoagulant use [5].

Meta‐analyses of WS have demonstrated that malignancies account for approximately two‐thirds of cases, with renal angiomyolipoma representing the most common benign etiology and renal cell carcinoma the most common malignant cause. Vascular disorders, including polyarteritis nodosa, renal artery aneurysms, and pseudoaneurysms, contribute to 20%–30% of the cases [5]. Infectious and inflammatory conditions, cyst rupture, and hydronephrosis represent less frequent causes. Rare associations have been described with anticoagulant exposure, hemophilia, post‐puerperal states, and toxic envenomation [6–10].

In our patient, DIC was excluded based on preserved coagulation parameters and absence of laboratory evidence of consumptive coagulopathy. Contrast‐enhanced abdominal CT and ultrasonography did not demonstrate renal masses, angiomyolipoma, or detectable vascular malformations. While the first episode occurred during ASA therapy, recurrence in the absence of aspirin exposure suggests that antiplatelet therapy alone was unlikely to be the sole precipitating factor; nevertheless, ASA remains a potential confounder for the initial event.

HU has been associated with cutaneous toxicity and vasculopathic manifestations, including leukocytoclastic vasculitis and vasculitic ulcerations. Case reports have described immune‐mediated vascular injury temporally related to HU exposure, suggesting potential endothelial toxicity and immune complex–mediated mechanisms. These observations provide biological plausibility for drug‐associated vascular fragility in susceptible patients [11].

The vasculitic hypothesis warrants careful consideration. The patient developed urticarial and petechial skin lesions accompanied by positive antinuclear antibodies (ANA, 1:160) and reduced C3 levels, findings that may be suggestive of an immune‐mediated small‐vessel process. C‐reactive protein and erythrocyte sedimentation rate were not significantly elevated, and there were no clinical signs of active infection. Urinalysis revealed macroscopic hematuria without dysmorphic erythrocytes, cellular casts, or significant proteinuria, arguing against clinically significant glomerulonephritis. Complement levels were not serially monitored; nevertheless, the isolated reduction in C3 raised suspicion for complement‐mediated immune activation.

A skin biopsy could not be performed due to the patient’s deteriorating clinical condition, active bleeding, and increased procedural risk. Therefore, histopathologic confirmation of leukocytoclastic vasculitis was not feasible. The differential diagnosis of the cutaneous findings includes drug eruption, small‐vessel vasculitis, Plt‐related purpura in the setting of MF, and less likely an infection‐related rash. However, the temporal association with HU exposure and the concomitant immune abnormalities support the possibility of a drug‐related immune mechanism.

Importantly, the first hemorrhagic episode occurred while the patient was receiving low‐dose ASA, which represents a potential confounder. Although ASA is widely used for thrombosis prevention in myeloproliferative neoplasms, antiplatelet therapy has only rarely been associated with spontaneous renal hemorrhage and may have contributed to the initial bleeding event. In this patient, ASA was initiated as part of standard management for thrombotic risk reduction in MF. However, the recurrence of bilateral hemorrhage after discontinuation of ASA suggests that antiplatelet therapy alone is unlikely to fully explain the clinical course.

HU has been associated with various vasculopathic and dermatologic toxicities, including leg ulcers, dermopathy, and immune‐mediated vascular injury. Proposed mechanisms include endothelial toxicity, immune complex deposition, and chronic inflammatory endothelial dysfunction. Although renal hemorrhage has not previously been reported in association with HU therapy, drug‐induced vascular fragility or immune‐mediated endothelial injury may theoretically predispose patients to spontaneous bleeding in susceptible vascular beds.

HU has been associated with cutaneous toxicity and vasculopathic manifestations. The temporal association between HU exposure, development of immune abnormalities (positive ANA and low C3 levels), purpuric lesions, and recurrent hemorrhage suggests a plausible immune‐mediated mechanism of vascular injury.

A structured literature search was performed in MEDLINE (PubMed) from database inception through October 15, 2025. The following search strategy was used: (“Wunderlich syndrome” OR “perirenal hemorrhage” OR “subcapsular renal hematoma”) AND (“hydroxyurea” OR “myeloproliferative neoplasm” OR “myelofibrosis”). No filters were applied for study design, and only articles published in English were included. Reference lists of relevant articles were manually screened to identify additional reports. Inclusion criteria comprised case reports, case series, and observational studies describing spontaneous renal or perirenal hemorrhage associated with HU or occurring in patients with myeloproliferative neoplasms. Articles describing traumatic renal hemorrhage or postprocedural hematoma were excluded. This search did not identify any previously published cases of hydroxyurea‐associated WS. To contextualize the novelty of the present case, previously reported WS cases related to hematologic conditions, anticoagulant or antiplatelet exposure, and vascular injury are summarized in Table 2.

**TABLE 2:** Previously reported Wunderlich syndrome cases in hematologic and vascular contexts.

Author (ref)	Underlying condition	Drug/trigger	Proposed mechanism	Outcome/notes
Medda et al. [2]	Mixed etiologies	—	Spontaneous renal hemorrhage leading to hemorrhagic shock	Demonstrates potential for severe clinical presentation
Zhang et al. [5]	Meta‐analysis (neoplastic and vascular causes)	—	Renal neoplasms (AML, RCC), vascular diseases	Malignancy identified as most common etiology
Kim et al. [7]	28 case series	—	Various spontaneous causes	Variable clinical outcomes
Mariolis‐Sapsakos et al. [6]	Renal cyst rupture	—	Structural cyst rupture	Benign non‐neoplastic etiology
Igarashi et al. [9]	Chronic hemodialysis	Anticoagulation during dialysis	Anticoagulant‐related bleeding	Non‐neoplastic cause of WS
Ruiz and Saltzman [10]	Post‐ESWL	Aspirin	Antiplatelet‐associated bleeding	Rare ASA‐associated bilateral renal hemorrhage
Senthilkumaran et al. [8]	Russell’s viper envenomation	Venom‐induced coagulopathy	Toxic endothelial and coagulation‐mediated injury	Rare non‐neoplastic trigger
Present case	Primary triple‐negative myelofibrosis	Hydroxyurea	Probable drug‐associated vascular injury (Naranjo score: probable)	To our knowledge, first reported WS temporally associated with hydroxyurea

Although causality cannot be definitively established, the temporal association, recurrence pattern, immunologic findings, and exclusion of common etiologies support a biologically plausible association between HU and WS in this patient.

To further assess causality, a structured evaluation using the Naranjo Adverse Drug Reaction Probability Scale was applied. The temporal relationship between hydroxyurea initiation and hemorrhage (+2), recurrence during continued exposure (+2), absence of other plausible causes (+1), and improvement after drug discontinuation (+1) yielded a total score of 6, corresponding to a “probable” adverse drug reaction. Although this scale has limitations in single‐case reports, the score supports a probable association between HU and the observed hemorrhagic event.

Clinically, WS may follow a self‐limited course but can also result in severe hemorrhage and hemodynamic compromise. In hemodynamically stable patients, conservative management—including intravenous fluid resuscitation, red blood cell transfusion when indicated, and discontinuation of anticoagulant or antiplatelet agents—is often appropriate. In cases with active contrast extravasation or persistent hemodynamic instability, interventional or surgical approaches such as transarterial embolization, partial nephrectomy, or radical nephrectomy may be required [12].

## 4. Conclusion

WS is a rare but potentially life‐threatening condition. While malignancies and vascular disorders remain the most common causes, clinicians should also consider potential drug‐associated cases. To our knowledge, this is the first reported case of WS temporally associated with HU therapy in MF. Early recognition and prompt evaluation of HU‐treated patients presenting with flank pain or hematuria are essential.

## Funding

This research did not receive any specific grant from funding agencies in the public, commercial, or not‐for‐profit sectors.

## Ethics Statement

Written informed consent for publication of this case report and accompanying images was obtained from the patient prior to her death. All identifying information has been removed. The study was conducted in accordance with the Declaration of Helsinki.

## Conflicts of Interest

The authors declare no conflicts of interest.

## Data Availability

The data used in this review are derived from previously published studies. No new data were generated or analyzed during the current study.
